# Complete Genome Sequence of *Methylobacterium populi* P-1M, Isolated from Pink-Pigmented Household Biofilm

**DOI:** 10.1128/genomeA.00458-16

**Published:** 2016-06-16

**Authors:** Tomohiro Morohoshi, Tsukasa Ikeda

**Affiliations:** a Department of Material and Environmental Chemistry, Graduate School of Engineering, Utsunomiya University, Utsunomiya, Tochigi, Japan; b JST, CREST, Kawaguchi, Saitama, Japan

## Abstract

*Methylobacterium populi* P-1M is isolated from the pink-pigmented household biofilm. Here, we present the complete genome sequence of P-1M, consisting of one chromosome of 5,705,640 bp and five plasmids of 64,864 bp, 59,879 bp, 42,569 bp, 41,417 bp, and 29,506 bp.

## GENOME ANNOUNCEMENT

The genus *Methylobacterium* is one of the dominant bacteria found in pink-pigmented household biofilms (1–3). Since many *Methylobacterium* strains exhibit higher tolerance to stress or chlorine, it would be quite laborious to remove already-established pink-pigmented biofilms (2). The bacterial interactions among the *Methylobacterium* species in household biofilms have not been clearly elucidated. In a previous study, we isolated 16 *Methylobacterium* strains from pink-pigmented household biofilms (3). Although all isolates formed low-level biofilms, the amount of the biofilms formed by strain P-1M was significantly increased by coculturing with the other *Methylobacterium* strains (3). A BLAST search revealed that the 16S rRNA sequences of P-1M showed identity to those of *Methylobacterium populi* type strain BJ001 (98.9%). *M. populi* BJ001 has been isolated from internal poplar tissues (4). The complete genome sequences of *M. populi* BJ001, which contained one chromosome and two endogenous plasmids, have been deposited in the DDBJ/ENA/GenBank database (accession numbers CP001029 to CP001031). In this study, we determined the complete genome sequence of *M. populi* P-1M.

Sequencing was performed on a Pacific Biosciences (PacBio) RS instrument (Pacific Biosciences, Menlo Park, CA) using libraries prepared with the SMRTbell template prep kit 1.0 (Pacific Biosciences) by TaKaRa Bio (Mie, Japan). We produced 208,290 reads with an average read length of 8,324 bases. The total number of sequenced bases is 1,733,978,071. The sequencing reads were assembled using the PacBio SMRT Analysis software version 2.2.0 (5). These reads were assembled into 6 large contigs. Prediction of putative coding sequences and gene annotation were done using the Microbial Genome Annotation Pipeline (http://www.migap.org/). Briefly, coding sequences were predicted by the combined use of MetaGeneAnnotator (6), RNAmmer (7), tRNAscan (8), and BLAST (9).

The genome of P-1M includes one chromosome and five plasmids. The one complete circular chromosome was 5,705,640 bp in size, with a G+C content of 69.23%. The five endogenous plasmids were 64,864 bp in size with a G+C content of 66.85% (named pMPPM01), 59,879 bp in size with a G+C content of 64.40% (named pMPPM02), 42,569 bp in size with a G+C content of 64.80% (named pMPPM03), 41,417 bp in size with a G+C content of 62.47% (named pMPPM04), and 29,506 bp in size with a G+C content of 64.18% (named pMPPM05). The chromosome contains 5,399 protein-coding genes, five rRNA operons, and 59 tRNA genes. The five plasmids, pMPPM01, pMPPM02, pMPPM03, pMPPM04, and pMPPM05, contain 63, 63, 40, 41, and 32 protein-coding genes, respectively. *M. populi* type strain BJ001 contains two endogenous plasmids. However, the sequences of the five plasmids from P-1M did not show significant similarity to those of two plasmids from BJ001.

### Nucleotide sequence accession numbers.

The complete genome sequence of *M. populi* P-1M has been deposited in the DDBJ/ENA/GenBank databases under accession numbers AP014809 (chromosome), AP014810 (plasmid pMPPM01), AP014811 (pMPPM02), AP014812 (pMPPM03), AP014813 (pMPPM04), and AP014814 (pMPPM05).
